# Diametrical diseases reflect evolutionary-genetic tradeoffs: Evidence from psychiatry, neurology, rheumatology, oncology and immunology

**DOI:** 10.1093/emph/eov021

**Published:** 2015-09-09

**Authors:** Bernard J. Crespi, Matthew C. Go

**Affiliations:** ^1^Department of Biological Sciences;; ^2^Department of Archaeology, Simon Fraser University, 8888 University Drive, Burnaby, BC, Canada V5A 1S6; ^3^Present address: Department of Anthropology, University of Illinois at Urbana-Champaign, 109 Davenport Hall, 607 S Mathews Avenue, Urbana, IL 61801, USA.

**Keywords:** tradeoffs, disease risk, pleiotropy, polygenic disorders

## Abstract

Tradeoffs centrally mediate the expression of human adaptations. We propose that tradeoffs also influence the prevalence and forms of human maladaptation manifest in disease. By this logic, increased risk for one set of diseases commonly engenders decreased risk for another, diametric, set of diseases. We describe evidence for such diametric sets of diseases from epidemiological, genetic and molecular studies in four clinical domains: (i) psychiatry (autism vs psychotic-affective conditions), (ii) rheumatology (osteoarthritis vs osteoporosis), (iii) oncology and neurology (cancer vs neurodegenerative disorders) and (iv) immunology (autoimmunity vs infectious disease). Diametric disorders are important to recognize because genotypes or environmental factors that increase risk for one set of disorders protect from opposite disorders, thereby providing novel and direct insights into disease causes, prevention and therapy. Ascertaining the mechanisms that underlie disease-related tradeoffs should also indicate means of circumventing or alleviating them, and thus reducing the incidence and impacts of human disease in a more general way.

## INTRODUCTION

A tradeoff exists between two phenotypes or genotypes when a benefit in one context entails a cost in another. Tradeoffs are caused by physical laws and biological constraints that limit ability to respond optimally to each of multiple fitness-related challenges [1]. As such, tradeoffs necessarily cause deviations from adaptations, considered as phenotypes that represent fully optimal fits to the environment that would lead to maximization of fitness.

For humans, deviations from adaptation can be conceptualized in terms of departures from good health, which manifest in risks and symptoms of disease. Tradeoffs are indeed, by this simple logic, fundamental evolutionary causes of human disease risks [2]. But what are the opposing selective pressures, and what is trading off with what to mediate liabilities to human disease?

**Figure 1.**
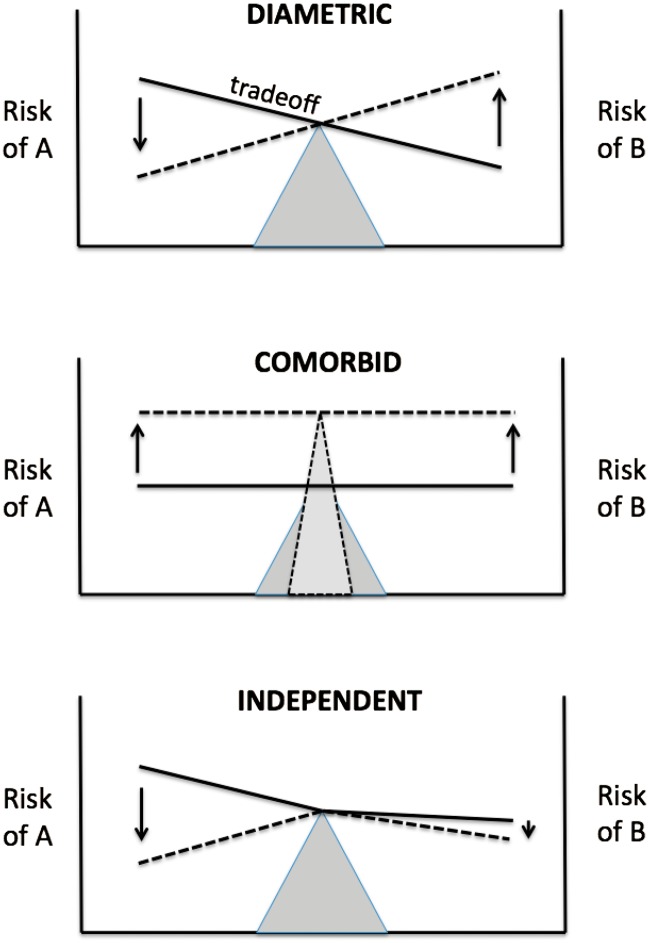
Three conceptual models for the relationship between two sets of diseases. Risks of diseases A and B are represented by the heights of the horizontally oriented lines, at the beginnings and ends of the arrows. Under the diametric model, sets of diseases are inversely related to one another due to tradeoffs between them, such that increases in risk of one disease result in decreases in risk of the other (as shown by the opposing vertical arrows). Under the comorbid model, diseases are positively associated due to common causes, such that increases in one set are linked with increases in the other. Under the independent model, sets of diseases are uncorrelated in risk and prevalence, because they lack shared causes; one example of possible independent responses to perturbation is shown

We suggest that human disease risks influenced by tradeoffs are commonly diametric, i.e. opposite to one another, such that sets of diseases often come in inversely associated pairs. Diametric diseases, defined here as sets of diseases that show opposite patterns in their causation, phenotypes and prevalence patterns, contrast sharply with diseases that are comorbid (positively associated) or independent of one another, in that under the diametric model increased risk of one set of diseases, in an individual or population, necessitates decreases in another (Fig. 1). Diametric patterns follow, in part, from the simple observation that biological systems may vary, and be perturbed, in two opposite directions, toward more activity versus less activity, higher expression versus less expression, larger versus smaller or earlier versus later. Relative extremes of such variation, due to effects of genetic and environmental variation, may then manifest in increased risks of diametric diseases.

Natural selection is expected to play important roles in the evolution of diametric disease risks, in four main ways. First, long-term selective, evolutionary trajectories generate the potential for diseases that involve alterations in the phenotypes and genotypes under selection. For example, the evolution of large human brain size has generated liability to microcephaly (much-reduced brain size) through losses of function in the evolved brain-growth system; gains of function are also possible in this evolved developmental-genetic system, leading to macrocephaly (enlarged brain size). Second, positive selection for beneficial phenotypes and genotypes may pleiotropically generate deleterious, disease-related effects, in phenotypes that trade off with them. Pleiotropy is indeed regarded as a universal mode of gene action [3], and it commonly manifests in tradeoffs [4]. Third, two selectively beneficial traits, such as strength and flexibility, or social and non-social skills, may trade off with one another, with consequences that are mediated by the form and strength of selection and the genetic bases of the traits. Fourth, disease itself represents a cause of selection, whose strength depends on its frequency and effects on survival and reproduction. In this context, ‘diseases’ can be regarded as disorders of function or structure involving particular bodily locations or cell types that grade more or less continuously from severe to mild in their effects. These roles of selection, though important for the evolution of disease risks, have seldom been analysed directly or quantified in this framework, so often remain largely conjectural.

**Figure 2.**
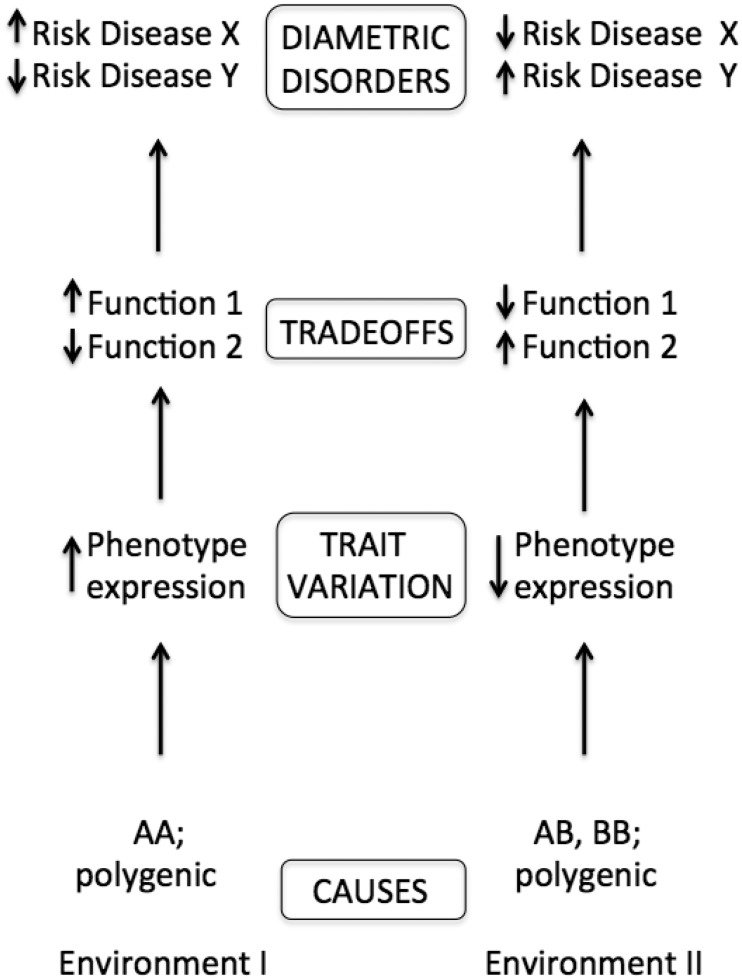
Depiction of the causes of diametric diseases and their mediation by tradeoffs. Genetic and environmental variation or perturbations cause variation in phenotypes with pleiotropic functions that tradeoff with one another, and variation in such functions affects risks of disease. For example, a genetically based lower threshold for apoptosis (programmed cell death) after DNA damage would shift the tradeoff between the benefits of cell retention and the costs of retaining damaged cells, which could increase risks of neurodegenerative diseases but decrease risks of cancer

By the diametric model, genetically or environmentally mediated increases in one function, or perturbations in one direction, should, given tradeoffs, increase risk of one set and form of diseases, whereas a diametric set and form of diseases should ensue from the opposite direction of variation (Fig. 2). What is especially interesting about diametric disorders is that, for each set of opposing conditions, higher risk for one set of diseases involves lower risk for the other [5]. As a result, determining the genetic and environmental causes of one class of diseases directly informs us about factors that protect against another class of diseases. Such protection should provide novel insights into preventatives and therapies.

In this article, we describe evidence salient to the diametric disease hypothesis from four major domains of human disease, (i) psychiatry (autism spectrum vs psychotic-affective spectrum disorders), (ii) rheumatology (osteoarthritis (OA) vs osteoporosis (OP)), (iii) oncology and neurology (cancer vs neurodegenerative disorders) and (iv) immunology (autoimmunity vs infectious disease). For each example, we describe the relevant diseases and their associations with human-evolved adaptation, provide a narrative review of empirical research findings salient to the diametric disease hypothesis, describe hypotheses and evidence regarding roles of selection in risks and effects of the diseases, and make predictions for future studies that follow from the evidence available to date. We also discuss how mechanisms that underlie the presence and strength of some tradeoffs may be subject to medical intervention, to alleviate risks from both diametric sets of diseases.

## AUTISM VERSUS PSYCHOTIC-AFFECTIVE DISORDERS

Autism, which refers here to autism spectrum disorders, is defined by deficits in social behavior combined with the presence of restricted non-social interests and repetitive behavior, with usual diagnosis in early childhood. Psychotic-affective spectrum conditions include schizophrenia, bipolar disorder, depression, borderline personality disorder, dissociative disorders and related conditions, all of which partially share a suite of symptoms including dysregulated and overly negative emotionality, hallucinations, delusions and other reality distortions, overly developed and arbitrary salience of perceptions, overly mentalistic (socially focused) cognition, mania and dissociation of normally integrated thoughts, memories and sense of identity [6–9]. Both autism and psychotic-affective conditions (with most study focused on schizophrenia) show substantial heritability, with effects from both common single-nucleotide polymorphisms of small effect and rare variants (such as copy-number variants) of large effect [10], and both sets of conditions grade in symptom and phenotype expression smoothly from normalcy to severe.

Autism was originally defined by Eugen Bleuler, ∼100 years ago, as a symptom of schizophrenia that involves social withdrawal [5]. In the 1940s, Kanner and Asperger adopted the term to refer to what they conceptualized as a separate disorder, with early-childhood onset. Kanner struggled to convince psychiatrists that autism was fundamentally different from schizophrenia, and for many years autism was considered by most practitioners as a form of schizophrenia with childhood onset. Kolvin [11] demonstrated that autism exhibited early-childhood onset, distinct from the usual adolescent or young-adult onset of schizophrenia, and subsequent studies have clearly differentiated the conditions (including a clause excluding dual diagnosis in the DSM).

Epidemiological studies on overlap of autism and schizophrenia diagnoses within individuals, over time, or within families, have produced highly variable results, ranging from 0% to ∼50% overlap [12], which have been attributed to false-positive childhood diagnoses of premorbidity to schizophrenia (symptoms expressed prior to diagnosis) as autism spectrum disorders, as well as diagnostic errors more generally [13–17]. The topic of overlap between autism and schizophrenia thus remains controversial.

Crespi and Badcock [18] hypothesized that autism and psychotic-affective conditions represent diametric disorders, with mechanistic and non-social cognition (cognition focused on non-social, rule-based systems and asocial sensory perceptions) increased in autism and reduced in psychotic-affective conditions, and mentalistic, social cognition (cognition focused on sociality, self-conceptions and self-other relations and empathic connections) increased in psychotic-affective conditions but decreased in autism (reviews in [19, 20]). By this hypothesis, risk and symptoms of psychotic-affective conditions derive from dysregulated overdevelopment of phenotypes that are unique or highly elaborated on the human lineage, including language, complex social relationships, sense of self, social-causal thinking, social striving and empathy. In contrast, autism spectrum condition phenotypes are mediated by underdevelopment of such traits and associated overdevelopment of non-social, mechanistic, abstract and perceptual foci, interests and abilities.

Tradeoffs between social and non-social interests and abilities, and between risks and phenotypes of autism and psychotic-affective conditions, are indicated by three main lines of evidence: social–nonsocial tradeoffs, diametric risk and protection and diametric phenotypes.

### Evidence for social–nonsocial tradeoffs

Among individuals with autism, and among neurotypical individuals, social skills tend to tradeoff with non-social skills, such as visual-spatial abilities, such that individuals with reduced social skills tend to exhibit increased non-social abilities (Table 1). By contrast, higher pedigree-based genetic liability to schizophrenia shows a strong correlation with better verbal skills relative to visual-spatial skills [33]. These findings suggest that tradeoffs exist between social and non-social abilities, and, more importantly, that autism and schizophrenia are associated with extremes of tradeoffs between social and non-social phenotypes. This conceptualization of autism dovetails closely with Baron-Cohen *et al.*’s [34, 35] extensive findings that the expression of autism is increased by a combination of low empathizing (social-emotional interest, motivation and abilities) with high systemizing (non-social, physical-world and rule-based interest, motivation and abilities), especially among males, and that individuals with autism, especially males, commonly show enhancements in visual-spatial abilities compared with neurotypical individuals [36–38]. Conversely, high empathizing combined with low systemizing has been linked with dimensional expression of paranoia and mania among neurotypical females [39], and cognitive-empathic skills, such as reading emotions from eyes, are increased over normal among females with borderline personality disorder or mild depression [19, 40]. Empathizing and systemizing are indeed negatively correlated with one another in some studies [22–24], though not in others [41–43]. Further evidence of tradeoffs comes from studies that compare individuals in technical compared with non-technical training or professions; these studies demonstrate lower empathizing and higher systemizing, and higher levels of autism in close relatives, associated with technical interests and skills, but higher empathizing and lower systemizing, as well as higher levels of psychotic-affective conditions in close relatives, associated with non-technical interests and skills [44–46].

**Table 1.** Evidence regarding diametric correlates between social and non-social skills

Task performance associations	References
Verbal skills negatively correlated with visual-spatial skills after adjustment for general intelligence	[21]
Empathizing Quotient test scores negatively associated with Systemizing Quotient test scores	[22–24]
Empathizing Quotent scores negatively correlated with Mental Rotation test scores	[25]
Measure of social interest and abilities negatively correlated with Mental Rotation test scores	[26]
False-belief task (theory of mind (ToM)) performance negatively correlated with Embedded Figures test performance	[27]
Social abilities negatively correlated with Embedded Figures test performance	[28, 29]
Social abilities negatively correlated with Raven’s Matrices test performance	[30]
‘Reading the Mind in the Eyes’ test performance negatively correlated with Embedded Figures test performance	[31, 32]

Considered together, these data suggest that tradeoffs between non-social and social cognition can mediate the expression of autistic compared with psychotic-affective phenotypes, with notable differences between the sexes. However, few studies have tested directly for cognitive tradeoffs, and the expression of such tradeoffs may depend on levels of overall cognitive resources: for example, Johnson and Bouchard [21] showed that verbal and visual-spatial intelligence are negatively correlated only after statistical adjustment for general intelligence. Additional studies that evaluate social and non-social abilities, in relation to both autistic and psychotic-affective psychological phenotypes and diagnoses, are required to evaluate the degree to which these psychiatric conditions reflect, in part, extremes of tradeoffs.

### Evidence for diametric risk and protection

If autism and psychotic-affective conditions represent diametric disorders, then they should exhibit diametric patterns of risk and protection, with factors that increase risk for one disorder protecting against the other. One of the most-penetrant risk factors for schizophrenia is a deletion of ∼50 genes (leading to haploidy) at chromosomal region 22q11.2; by contrast, duplications of this same region (leading to triploidy) both protect against schizophrenia and increase risk of autism [47] (Table 2). Comparable opposite risk patterns (where deletions are associated with one disorder, and duplications are associated with the other disorder) exist for the copy-number variant loci 1q21.1, 15q11.2 and 16p11.2 [16–19] (Table 2), although protective effects have not been tested. Similarly to this copy-number data, high birth size (weight or length) has been associated with higher risk of autism but lower risk of schizophrenia spectrum disorders, and low birth size has been associated with lower risk of autism but higher risk of schizophrenia spectrum disorders [53]. Finally, congenital blindness appears to provide complete protection against schizophrenia [59, 60], and it also represents a clear risk factor for autism spectrum phenotypes [57], both for reasons that require additional investigation. These diverse but convergent findings strongly support the diametric model, and they should motivate further studies that jointly evaluate risk and protection for causal factors underlying autism and psychotic-affective conditions.

**Table 2.** Evidence regarding diametric genetic risk factors, phenotypes and correlates of autism spectrum and psychotic affective spectrum conditions

Trait	Autism spectrum	Psychotic-affective spectrum	Comments
Copy-number variants, 22q11.2 (deletions compared with duplications of chromosomal region)	Duplications of 22q11.2 increase autism risk [16, 47]	Duplications of 22q11.2 decrease schizophrenia risk; deletions of 22q11.2 greatly increase schizophrenia risk [47]	Deletions of 22q11.2 suggested to increased autism spectrum disorder risk but pattern not found in autism spectrum disorder copy-number variants cohorts [16]; presence of increased autism risk in 22q11.2 deletions is controversial
Copy-number variants, 1q21.1	Duplications of 1q21.1 increase autism risk, increase head size [16, 48]	Deletions of 1q21.1 increase schizophrenia risk, reduce head size [48, 49]	Deletions may increase autism risk or be false positive [16]
Copy-number variants, 16p11.2	Deletions of 16p11.2 increase autism risk, increase head size [50]	Duplications of 16p11.2 increase schizophrenia risk, reduce head size [49, 50]	Duplications may increase autism risk or be false positive [16]
Copy-number variants, 15q11.2	Duplications of 15q11.2 (BP1-BP2 region) increase autism risk [51]	Deletions of 15q11.2 (BP1-BP2) increase schizophrenia risk [49]	Deletions and duplications of *CYFIP1*, a key gene in this CNV region, cause opposite alterations to dendritic spine complexity [52]
Birth size (weight, length)	Smaller birth size associated with lower autism risk; larger size associated with increased autism risk [53]	Larger birth size associated with lower schizophrenia risk; smaller size associated with increased schizophrenia risk [53]	Each of the patterns of risk has been replicated across many other studies
Brain size	Larger brain size in children with autism [54, 55]	Smaller brain size in schizophrenia [56]	Autism involves faster brain growth in early childhood, in particular
Congenital blindness	Congenital blindness increases autism risk, phenotypes [57, 58]	Congenital blindness protects against schizophrenia [59, 60]	
Sensory abilities	Sensory abilities increased in autism [61–68]	Sensory abilities decreased in schizophrenia; sensory deprivation induces features of psychosis [69–76]	Strong, highly consistent pattern in schizophrenia; substantial although somewhat mixed evidence in autism
N-methyl-D-aspartate (NMDA) receptor function (glutamate receptor) involved in synaptic plasticity	NMDA receptor hyperfunction in autism [77]	NMDA receptor hypofunction in psychosis, schizophrenia [77]	
Prepulse inhibition (initial stimulus reduces reaction to second stimulus)	Prepulse inhibition increased in autism [78, 79]	Prepulse inhibition decreased in schizophrenia [80]	Findings highly consistent for schizophrenia, variable for autism
Mismatch negativity (brain response to unexpected stimulus)	Mismatch negativity increased in autism [81]	Mismatch negativity decreased in schizophrenia [82, 83]	Findings highly consistent for schizophrenia, variable for autism
Mirror neuron system (system whereby same neurons are activated in perception of an action as in enacting it)	Mirror neuron system activation decreased in autism [84, 85]	Mirror neuron system activation increased in actively psychotic individuals with schizophrenia [86]	Same protocol used to measure mirror neuron function, in autism and schizophrenia [86]; other studies of schizophrenia usually show reduced activation [87] but do not involve actively psychotic subjects
Default mode system (brain regions active in stimulus-independent thought) activation	Default mode system activation reduced in autism, in association with reduced self-referential and imaginative cognition [88–91]	Default system overactivated in schizophrenia, in association with reality distortion and increased imaginative cognition [89]; also less deactivation of this system [92]	Some studies of autism show reduced deactivations of default system, that may be associated with reduced activation [89]; Immordino-Yang *et al.* [93] also contrast autism and schizophrenia as opposite with regard to the default network
Default mode connectivity	Reduced connectivity within default mode in autism [94, 95]	Increased connectivity within default mode in schizophrenia [96–98]	Some mixed results in both autism and schizophrenia but two reviews support opposite nature of the alterations [99, 100]
Brain connectivity	Increased local brain connectivity, decreased long-range connectivity, in association with early brain overgrowth [55]	Decreased local brain connectivity, increased long-range connectivity, in association with increased cortical thinning, in childhood-onset schizophrenia [55]	Findings based on review of neuroimaging findings [55]
Temporal-parietal junction activation	Temporal-parietal junction region shows reduced activation in autism, underlies mentalizing reductions [101, 102]	Temporal-parietal junction region shows increased activation in schizophrenia, underlies some psychotic symptoms [103, 104]	
Social motivation	Reduced social motivation in autism [105]	Increased social motivation in mania, hypomania [106, 107]	Motivation in general decreased in negative symptom schizophrenia, depression
Cognitive empathy (reading emotion from non-verbal cues)	Cognitive empathic abilities reduced in autism [108]	Some cognitive empathic abilities enhanced in borderline personality disorder and subclinical depression [40, 109]	Cognitive empathic abilities lower in schizophrenia, bipolar disorder and depression, in association with general cognitive deficits [110, 111]
Social emotion	Reduced social emotion in autism [112]	Increased social emotion expression in bipolar disorder and depression (e.g. guilt, shame, embarrassment, pride) [113, 114]	Reduced general expressed emotionality in negative symptom schizophrenia
Inattentional blindness (failure to recognize unexpected stimulus that is in plain sight)	Decreased inattentional blindness in autism [115]	Increased inattentional blindness in schizophrenia [116]	
Selectivity of attention	Overselective attention [117, 118]	Reductions in selective attention in schizophrenia and positive schizotypy [119, 120]	
Stroop task (test measuring selective attention, using words written in different colors) performance	Enhanced Stroop task performance in autism [121]	Decreased Stroop task performance in schizophrenia, by meta-analysis [122]	Results mixed for autism, highly consistent for schizophrenia
Iowa Gambling Task (test of decision-making impulsivity) performance	Enhanced Iowa Gambling Task (test of decision-making impulsivity) performance in high-functioning autism [123]	Reduced Iowa Gambling Task performance in schizophrenia, in most studies [124]	Results mixed for autism, consistent for schizophrenia
Susceptibility to rubber hand illusion (induced belief that rubber hand is one’s own)	Reduced susceptibility to rubber hand illusion in autism and in healthy high-ASD trait individuals [125–127]	Increased susceptibility to rubber hand illusion in schizophrenia [128]	Same general pattern also found for visual illusions, with some inconsistencies [129]
Word interpretation	Literal word interpretation, underinterpretation of social relevance, in autism [130]	Overinterpretation of word meaning and social relevance in schizophrenia [130]	
False memory induction	Decreased induction of false memories [131–133]	Increased induction of false memories associated with psychosis phenotypes [134–136]	Results somewhat mixed (some non-significant) for autism
Semantic memory network states	Semantic memory network states overly rigid in autism [137]	Semantic memory network states chaotic in schizophrenia [137]	
Working memory performance	Working memory deficits in autism [138]; extraordinary working memory enhancements in child prodigies, who score above autism range in Attention to Detail on Autism Quotient test, and exhibit high rates of autism in their families [139]	Large working memory deficits in schizophrenia; highly consistent finding [140, 141]	Findings of Ruthsatz and Urbach [139] would benefit from replication; areas of excellence in child prodigies notably overlap with those found in savantism in autism [142]
Reading abilities	Hyperlexia (precocious, fast reading, usually with poor comprehension) found predominantly in autism [63, 143, 144]	Dyslexia associated with schizophrenia and schizotypy [145–147]	Williams and Casanova [148] contrast autism and dyslexia for cortical microstructure
Decision making	More-deliberative decision-related processing in autism [149]	‘Jumping to conclusions’ associated with delusions in schizophrenia [150, 151]	
Bayesian perception model parameter values	Bias toward hypo-priors in Bayesian models of perception and cognition [152, 153]	Bias toward hyper-priors in Bayesian models of perception and cognition [152, 154]	
Inference of intentions of others	Reduced inference of intentions in autism [155]	‘Hyper-intentionality’ in schizophrenia and schizotypy [155–157]	Bara *et al.* [158] contrast autism and schizophrenia directly in this regard
ToM performance, ToM Storybooks test	Reduced ToM in autism spectrum children by ToM Storybooks test [159]	‘Hyper-Theory-of Mind’ in children with more psychotic experiences by ToM Storybooks test [160]	
ToM performance, MASC (movie for the assessment of social cognition) test	ToM abilities reduced in autism, using MASC test, due to combination of hypo-mentalizing, lack of mentalizing and hyper-mentalizing [161, 162]	ToM abilities reduced in association with positive symptoms of schizophrenia, using MASC test, due to hyper-mentalizing [163, 164]; hyper-mentalizing also found in borderline personality disorder using MASC [165]	
Salience of social and non-social stimuli	Reduced salience of social stimuli, and overly specific and inflexible salience of primary perceptual and non-social stimuli [166, 167]	Overdeveloped and arbitrary salience in prodrome and psychosis, mainly involving social phenomena [9, 168, 169]	
Perception of biological motion	Decreased perception of biological motion, entities, in autism; fail to see humans who are there [170]	Increased and false perception of biological motion, entities, in schizophrenia; see humans in random dots [171]	
Visual-spatial abilities	Selectively enhanced visual-spatial abilities in autism [172, 173]	Reduced visual-spatial skills, relative to verbal skills, positively associated with genetic liability to schizophrenia [174]; also see [175]	
Embedded Figures Test performance	Enhanced Embedded Figures Test performance among healthy individuals with more autistic traits [176]	Reduced Embedded Figures Test performance among healthy individuals with more positively schizotypal traits [176]	
Imagination and creativity	Reduced imagination and creativity in autism [177, 178]; review in (B. Crespi *et al.,* submitted for publication)	Increased imagination and creativity, in schizophrenia, schizotypy and bipolar disorder and in relatives [179–183]; review in (B. Crespi *et al.,* submitted for publication)	The literature relating psychotic-affective spectrum phenotypes and conditions to aspects of increased imagination and creativity is large and diverse; reduced imagination has been considered as a diagnostic criterion for autism
Pretend and social play in childhood	Reduced pretend play and social play in autism [184, 185]	Higher levels of dissociation, hallucination, psychotic-affective psychopathology associated with presence of childhood imaginary companions [186–189]	
Professions	Autism associated with technical professions in fathers, mothers and grandfathers [190–192]	Schizophrenia, schizotypy, bipolar disorder and depression associated with careers and interests in arts, humanities and literature [193, 194]	
College majors	Autism in family associated with technical college majors [46]	Bipolar disorder, depression in family associated with arts and humanities majors [46]	Insufficient data on schizophrenia for analysis, in this study
Socioeconomic status	Autism associated with higher socioeconomic status [195, 196]	Schizophrenia associated with lower socioeconomic status [197]	
Sex ratios	Autism shows strongly male-biased sex ratio [35, 198]	Considering incidence rates of schizophrenia (∼1%, with a male bias of ∼1.4:1), bipolar disorder (∼1%, with sex ratios about equal) and depression (∼15%, with a strong female bias of ∼2:1), these psychotic-affective conditions show an overall population-wide female bias [199–201]	

Adapted and extended from [19]. See [18] for citation off earlier and additional studies

### Evidence for diametric phenotypes from neurological, psychological, epidemiological and genetic data

If autism and psychotic-affective conditions represent diametric disorders, they should exhibit opposite phenotypes for correlates of the two sets of conditions, with neurotypical phenotypes being intermediate and ‘normal’. Table 2 presents a large set of variables for which such opposite patterns have been described. These findings support the diametric hypothesis, most notably with regard to underexpression versus overexpression of neurological, cognitive and behavioral traits that have apparently undergone evolutionary changes along the human lineage. The primary limitation of such findings is that few of the studies have collected data from individuals with autism, psychotic-affective conditions and controls using the same protocols, in the same paper; moreover, considerable heterogeneity exists in the consistency of results, especially for autism.

As noted earlier, some authors have claimed that autism and schizophrenia are similar, overlapping disorders, an alternative hypothesis that is in direct opposition to the hypothesis of diametric disorders described here [202]. These claims are, we believe, based on a small set of misconceptions, including (i) considering ‘social deficits’ as central to both autism and schizophrenia, without noting that such deficits can be due to extremely different, and opposite, cognitive alterations; (ii) considering overlaps in risk genes or copy-number variant loci as evidence of similarity, when the linkages may be due to alternative alleles or genotypes at a locus (or different loci entirely), or deletions versus duplications of the same copy-number region and (iii) not considering the expectation that some proportion of autism spectrum disorder diagnoses will represent false-positive diagnoses of premorbidity to schizophrenia (psychological and psychiatric problems in children who will later develop schizophrenia), especially given that ‘autism spectrum’ is the only available DSM diagnosis for children with social deficits [14–16, 203].

### Evolutionary considerations and clinical implications

The diametric hypothesis for autism and psychotic-affective conditions can provide insights into human cognitive evolution because it is evolutionary changes along the human lineage that provide scope and structure for psychiatric symptoms. As such, human brain evolution has involved enhancement and elaboration of social-cognitive phenotypes, and cognitive traits that show clear diametric phenotypes, such as human language [204], and imagination and creative cognition (B. Crespi *et al.,* submitted for publication), are expected to have evolved along a trajectory toward the cognitive phenotypes that are overdeveloped in psychotic-affective conditions. Schizophrenia ‘risk genes’ are, by this hypothesis, expected to include genes that mediate social cognition, imagination and language, with ‘risk alleles’ commonly mediating enhanced performance [205] or tradeoffs [20]; these considerations are generally concordant with the ‘social brain’ hypothesis for human brain enlargement and elaboration [206], and connect it with risks for psychiatric disorders involving social cognition and behavior [207]. In contrast, autism ‘risk genes’ may, at least in part, mediate enhanced performance in non-social tasks [208] that trade off with social abilities.

A primary clinical usefulness of the diametric model, with regard to autism and psychotic-affective conditions, is that it generates reciprocal illumination between the two sets of conditions, such that findings for one set of disorders immediately generates insights and novel research questions with regard to the others. Such insights extend directly to pharmacological and behavioral therapies; for example, antagonists for the GRM5 glutamate receptor have been developed to treat individuals with autism spectrum disorders [209, 210], whereas agonists for the same receptor are being developed and tested for schizophrenia [211]. Similarly, behavioral therapies for autism in young children commonly target enhancement of imaginative cognition [212] but therapy for psychotic-affective conditions focuses on reducing overly expressed and dysregulated social-imaginative cognition [213]. Systematic application of insights from the diametric model has the potential to guide research along novel and promising paths, with implications for both psychiatry and how human behavior has evolved.

## OA VERSUS OP

OA is classically defined as the breakdown of articular cartilage in highly mobile joints [214]. However, the disease also involves changes to bone. In particular, OA-afflicted joints undergo subchondral bone sclerosis (increased bone density under cartilage) and develop marginal osteophytic growths (bone spurs) [215] that represent major causes of morbidity. OP, by contrast, is characterized by the loss of bone and deterioration of internal bone microstructure and trabecular networks [216]. This loss of bone occurs through imbalance in bone homeostasis, whereby the rate of bone resorption exceeds that of deposition. OP increases the risk of fractures, especially at load-bearing sites such as the lower spine and hip. Although not fully understood, the etiologies of both diseases are widely accepted as multifactorial, with both environmental and genetic components playing important roles in pathophysiology [217, 218].

OA and OP have been hypothesized as inversely related for many years, originally based on clinical and epidemiological observations of patients with OA rarely being afflicted with OP, and vice versa [219–221]. Since these initial observations, considerable evidence salient to the diametric hypothesis has accumulated from clinical, epidemiological, molecular and genetic data (summarized in Table 3). We discuss this evidence in detail as it has not been comprehensively reviewed elsewhere.

**Table 3.** Evidence regarding diametric epidemiology, risk factors, genotypes, phenotypes and correlates between OA and OP

Phenotype or genotype	Patterns in OA and OP	References
Epidemiology	Epidemiological studies suggest those afflicted with OP may have a reduced risk of or have protection against OA.	[222–224]
Epidemiology	Daughters of mothers with OA have a reduced risk of hip fractures, suggesting OA may protect against OP. Additionally, daughters of mothers with OA have increased peak bone mass at the hip.	[225, 226]
Risk factors, correlates	Apparent inverse relationship between risk factors of OA and OP, such as obesity and mechanical overloading for OA and low BMI or body weight and immobility for OP.	[227]
Risk factors, correlates	Inverse anthropometric phenotypes were observed between women with OA (more obese, had more fat, muscle mass and strength) and women with OP (more slender, had less fat, muscle mass and strength).	[228]
*TGF-β1* locus	C-allele of *TGF-β1* is more prevalent in Japanese women with spinal osteophytosis (bone spurs, indicative of OA) and much lower in those with OP.	[229]
*LRP5* locus	Point mutation in the *LRP5* gene causes autosomal dominant high bone mass, a trait of OA, while loss of function of the gene causes OP-pseudoglioma, lower BMD and increased incidence of bone fractures.	[230–232]
*FRZB* locus	Lories *et al.* [233] found a differential association of alleles of the Arg200Trp single-nucleotide polymorphism in the WNT antagonist *FRZB* gene between patients with hip OA compared with patients with OP. Partial replication, for OA, reported by Rodriguez-Lopez *et al.* [234] but other studies did not demonstrate significant effects.	[233–236]
WNT pathway activation	Canonical WNT pathway activation leads to increased bone mass and strength, a characteristic of OA, while inhibition of the pathway leads to decreased bone mass and strength, a characteristic of OP.	[237]
WNT pathway expression and alleles	WNT activity increased and WNT pathway genes upregulated in OA patients compared with OP patients. However, no significant allelic differences were found between OA and OP patients, for 24 SNPs in genes that showed differential expression between OA and OP.	[238–240]
WNT pathway expression and alleles	Microarray gene expression profiling suggests alterations in the WNT and TGF-β pathways of OA patients versus OP patients and controls. Furthermore, deregulation of WNT and TGF-β signaling pathways was demonstrated in bone and osteoblasts from patients with hip OA. Samples from patients with OP were not studied.	[241–242]
Gene expression	Several genes involved in apoptosis and osteogenesis show higher expression in OA patients versus OP patients, suggesting less transcriptional activity in OP.	[243]
Gene expression	Genome-wide analysis of trabecular bone samples from OA and OP patients revealed inversely methylated and expressed genes between the two groups, especially for genes involved in cell differentiation and skeletal embryogenesis.	[244]
Bone characteristics	Increased bone turnover and reduced trabecular bone quality were observed in patients with OP, compared with retarded bone turnover and increased trabecular bone quality in patients with OA.	[245, 246]
Bone characteristics	Significant bone microstructural differences including bone volume fraction, trabecular thickness and mean roundness were found between postmenopausal women with OA compared with postmenopausal women with OP. Control group not used due to difficulty in finding women fully unaffected by either condition. The results ‘convincingly support the hypothesis that there might be an inverse relationship between OP and OA’.	[246]
Bone characteristics	BMD significantly higher in OA and significantly lower in OP versus controls in both men and women from epidemiological studies.	[247–252]
Bone characteristics	Levels of osteocalcin were higher in OA groups versus OP groups, suggesting higher osteoblastogenesis (growth of cells from which bone develops) in OA.	[253–255]
Bone characteristics	Adiponectin, lipoprotein lipase and hormone sensitive lipase, which are associated with higher osteoblastogenesis and lower osteoclastogenesis, are higher in OA patients versus OP patients. Authors ‘conclude that OP bone tissue exhibits lower osteoblastogenesis, higher osteoclastogenesis and lower troglyceride metabolism compared with OA or healthy controls’.	[256]

BMD, bone mineral density; BMI, body mass index

### Clinical and epidemiological evidence

Bone mineral density (BMD), which represents a key aspect of both OP and OA, shows clear contrasting patterns between these two diseases. Thus, whereas OP is fundamentally characterized on the basis of reduced BMD [257], significant increases in BMD have been associated with OA [247]. Similarly, higher BMD measures have also been found in women with OA affecting the hands, knees and lumbar vertebrae [249]; the same association was also found in two other studies of postmenopausal women [250, 252]. In a comparison between women with OA and women with OP, the OA group has also been shown to exhibit significantly higher BMD scores at four anatomical sites [251]. In a comparable study of elderly men, significantly higher BMD values at the hip and lumbar joints were found in men with OA compared with controls [248]. These findings convergently support the hypothesis that high BMD is associated with OA and low BMD with OP.

Bone turnover, or the rate at which new bone is made and old bone is destroyed, is a crucial determinant of the processes that cause OA and OP. Newly deposited bone requires time to strengthen and mineralize. However, in OP, bone turnover rates are increased, and higher production and activity of osteoclasts (which remove bone) relative to osteoblasts (which generate bone) result in the net loss of bone characteristic of OP [258]. OP can thereby result from either the overproduction of osteoclasts, the underproduction of osteoblasts, or some combination of the two processes [219, 259]. The net result is a decrease in both bone quality and quantity, with such imbalances in bone homeostasis increasing the risk of fractures in OP [260]. Inversely, bone remodeling is reduced in OA patients [219, 251], leading to bone overproduction. For example, in measuring bone turnover markers from serum and micro-CT imaging, Montoya *et al.* [245] found increased bone turnover and poorer trabeculae quality in OP patients compared with OA patients; they concluded that ‘bone microstructural changes in OA are opposite to those of OP’ [245:304]. Similar significant and diametric differences between patients with OP and OA have been found using high-resolution magnetic resonance imaging [246]. Considered together, these findings support the diametric hypothesis with respect to BMD and its correlates, which represent key manifestations of bone function and physiology.

Anthropometric phenotypes between OA and OP also show evidence of diametric patterns [228, 251]. Thus, on one hand, women with OP tend to be of shorter stature with less fat, muscle and strength. On the other hand, women with OA tend to be more obese with more fat, muscle and strength even when controlling for age and skeletal size. These patterns apparently derive, at least in part, from the fact that mesenchymal stem cells within bone marrow can develop into either bone forming osteoblasts or fat storing adipocytes [261], such that stem cell up- or down-regulation should affect both tissues.

The runt-related transcription factor 2 (*RUNX2*), together with its downstream target genes osterix and osteocalcin, is mainly responsible for regulating the patterns of osteoblast differentiation that give rise to these anthropometric patterns. In turn, the peroxisome proliferator-activated receptor γ *(PPARγ*) controls adipocyte differentiation together with its downstream target genes adiponectin, perilipin 2, angiopoietin-like 4 and fatty acid binding protein 4. Dragojevic *et al.* [253] found higher gene expression of RUNX2, osterix, osteocalcin, *PPARγ2* (a *PPARγ* isoform) and adiponectin in OA patients compared with OP patients, all of which suggests that osteoblastogenesis and adipogenesis are higher in OA. These results lend convergent support to the clinically observed increases in BMD and obesity phenotypes in OA and provide clear evidence of tradeoffs at the cell-differentiation pathway level.

The diametric hypothesis for OA and OP also predicts that the development of OA within a patient may protect against the development of OP. In support of this hypothesis, one study showed that the risk for femoral neck fractures was significantly reduced in both men and women with OA compared with controls and patients who had suffered hip fractures [222]. Moreover, elderly women who had previously suffered from fractures due to OP also exhibited lower OA-indicator measurements than those who had not suffered fractures [223].

Family-based studies have shown further evidence of diametric effects in OA compared with OP [219, 262]. For instance, the risk of hip fractures is significantly reduced in daughters of mothers with OA [225]. Additionally, peak bone mass in the hip of daughters with OA mothers is increased [226]. Twin studies have shown higher BMD in the femoral neck of individuals with hip joint osteophytes (bone spurs) versus their unaffected twins [263]. Considered together, and in the context of the substantial heritabilities of OA [264] and OP [259], these findings provide evidence of diametric risk underlain by polygenic effects.

### Molecular evidence: signaling pathways mediating OA and OP

As described earlier, OA and OP can be understood as resulting from imbalances in bone homeostasis, where the rates of bone production, maintenance and resorption are disturbed in either direction. These rates are regulated in large part by the wingless-type mouse mammary tumor virus integration site (WNT)/β-catenin or ‘canonical’ WNT signaling pathway, which modulates physiological processes in bones and cartilage and has been the focus of several research efforts into the diametric OA-OP hypothesis [237, 240, 241, 265, 266].

Altered regulation of the WNT pathway, or mutations of the genes involved, have been associated in several studies with OA and OP pathology. First, WNT activity is increased in bone samples and osteoblast cultures retrieved from patients with OA compared with those with OP-related hip fractures [240], with OA-associated upregulation of several WNT-pathway related genes (*BCL9, FZD5, DVL2, EP300, FRZB, LRP5* and *TCF7L1*). García-Ibarbia *et al.* [238] found decreased WNT activity in patients with OP hip fractures in comparison with OA patients, although allelic differences were not found for SNPs in a set of genes that were differentially expressed in OA versus OP. Papathanasiou *et al.* [239] reported significantly upregulated canonical WNT pathway genes in chondrocytes from OA patients, affirming the dual involvement of bone and cartilage in the disease pathology. Baron and Kneissel [237] attributed increased bone mass and strength to WNT activation, while the opposite was true for inhibition of this pathway. WNT/β-catenin signaling is essential for both osteoblast proliferation, and in some cases the downregulation of apoptosis. Additionally, osteoblasts (and their terminally differentiated forms as osteocytes) are signaled by the WNT pathway to produce osteoprotegerin (OPG), which suppresses production of osteoclasts [237, 267].

Osteoclast functioning is also regulated, in part, by RANKL-RANK (receptor activator of nuclear factor κB ligand) signaling downstream of the WNT pathway [268]. The RANK receptor (receptor activator of NF-κB) located on hematopoietic osteoclast progenitor cells and mature osteoclasts is activated by the RANK ligand RANKL. Successful RANKL-RANK binding initiates a signaling process that is necessary for the transformation of progenitor cells into osteoclasts, and in the activation of mature osteoclasts [269]. OPG, a decoy receptor of RANKL, inhibits RANKL-RANK binding, and thus discourages osteoclastic bone turnover [269]. Several studies have identified higher levels of OPG, and higher RANKL/OPG ratios, from serum, bone specimens and osteoblasts in OP patients compared with OA patients [251, 255, 270, 271]. RANKL knockout mice also exhibit severe osteopetrosis (extremely dense bone) [272], due to greatly reduced osteoclastic function. Indeed, bone remodeling rates and sites are increased in OP, and the RANKL-RANK pathway has been considered as an important target in pharmaceutical interventions of the disease [268, 269].

A third important pathway identified in bone homeostasis is transforming growth factor-beta (TGF-β) signaling. Member molecules of the relatively large TGF-β family play essential and multiple roles in embryonic and postnatal development, cell proliferations, differentiation, apoptosis and other processes. In bone, TGF-β signaling promotes the deposition of the extracellular matrix components Col1 and osteocalcin, which allows osteoblasts to complete their maturation [273]; in this context, osteocalcin levels have been used as a measure of osteoblastogenic activity [253]. Low serum levels of osteocalcin have been found in OP patients compared with OA patients [251, 254], while higher concentrations have been found in serum and mesenchymal cells of OA individuals compared with OP patients [251, 253, 255]. Such findings suggest increased bone formation in OA, which has been verified in clinical observations of the disease. The TGF-β pathway is also involved in cartilage and synovial tissue and has been implicated in several symptoms of OA such as cartilage degradation, osteophyte formation and low-grade synovitis [274]. Data from bone samples of the iliac crest in OA patients found increased concentrations of TGF-β and suggest reduced bone remodeling compared with OP [242, 275].

An important caveat regarding these results is that few studies have compared molecular or physiological phenotypes between OA patients, controls, and OP patients in the same analysis, in part because ethical considerations reduce availability of bone tissue samples from healthy individuals. Despite such limitations, Papathanasiou *et al.* [239] were able to obtain cartilage samples from patients without disease to use as controls; they found increased WNT activation through enhanced *LRP5* gene activity in OA versus controls. Similarly, Dragojevic *et al.* [256] obtained healthy control samples from autopsies, and found differences in osteoblastogenesis and osteoclastogenesis between groups (i.e. OA patients, OP patients and autopsy control samples) that were consistent with the diametric model. Further studies that compare both OA patients and OP patients with controls are clearly needed to provide the most direct tests of the molecular underpinnings of the diametric model in this context.

Considered together, these findings lend strong molecular support to the clinical and anthropometric observations of OP and OA phenotypes, indicating that decreased WNT activity (and comparable effects from the RANK and TGF-β pathways) engender decreased bone density and strength, whereas increased activity results in stronger bone but adverse effects on articular cartilage [265]. This tradeoff is thus mediated in large part by diametric patterns of pathway activity, such that higher activation leads to greater bone strength, which decreases risk of OP and fractures, but increases risk of OA, which causes pain and impairs mobility of joints.

### Genetic evidence for diametric risk for OA and OP

Studies of signaling pathways provide useful evidence regarding the diametric hypothesis for OA and OP in that they link the expression of clinical symptoms with sets of genes and proteins. Genes of research interest for these disorders have typically been those involved in bone homeostasis signaling pathways, especially the WNT and RANKL-RANK, and WNT-pathway associated genes have indeed been identified from genome-wide association studies (GWAS) of osteroporosis [276].

Loss of function experiments on the *LRP5* gene, a component of the WNT pathway, have shown increased cartilage degradation in instability-induced OA models in mice [277]. In humans, loss of function of the *LRP5* gene causes the recessive disorder Osteoporosis Pseudoglioma Syndrome, which involves severe juvenile OP and frequent fractures [231, 278]. By contrast, a non-synonymous point mutation (Gly171Val) in the *LRP5* gene results in autosomal dominant high bone density [230, 232], which may be caused by the product of the dickkopf gene DKK-1 ineffectively inhibiting *LRP5* receptors within the canonical WNT pathway [230]. This gene thus shows notable evidence of diametric effects on bone density from loss versus apparent gain of function.

The frizzle related protein B (*FRZB*) gene likewise shows evidence of an inverse, genetically based effect in OA compared with OP, in that for the Arg200Trp polymorphism in this gene, individuals with OA showed a higher frequency of the T allele than did individuals with OP, with controls intermediate in allele frequency between the two [233]. Experiments with mice knockouts for the *FRZB* gene also show increased WNT activity, resulting in symptoms of OA including cartilage damage, and bone thickening and stiffness ([279]; but see also [280]). By contrast, genetic association studies with other markers in this gene, focused on OA, have yielded mainly negative results [234–236, 281]. Conducting direct genetic tests of the diametric model for OA and OP necessitates a larger set of well-established risk loci, or comparisons of GWAS results.

### Evolutionary considerations and clinical implications

How do these considerations relate to trajectories of human evolution, with regard to bone density and associated phenotypes? Increased rates of OA and OP in humans, compared with other great apes, have been attributed to the evolution of bipedal locomotion, which changes range of motion for some joints, and increased stresses that can lead to spinal and other fractures [282–284]. Current industrialized and agricultural human populations, however, exhibit lower BMD than do great apes, fossil hominins and foraging human populations [284–286]. These differences can apparently be attributed to the recent adoption of more sedentary lifestyles in most human populations [286, 287], given that bone density peaks in early adulthood in direct relation to levels of biomechanical loading during the first part of the lifespan [288]. Risk for OP thus appears to now depend strongly on gene-by-environment interactions mediated by environmental novelty (sedentary behavior), which may also be expected, under the diametric disease model, to decrease risks of OA. Alexander [282] likewise describes observational evidence that joint ranges of motion are generally higher in two species of apes than in humans, which may be ascribed, in part, to upright posture and recent changes in human behavior that reduce joint motion. These findings suggest that risk of OA can be reduced through increased range of joint motion activity throughout the lifespan [282].

Recent human-evolutionary changes in BMD have been inferred by Medina-Gómez *et al.* [289], who used a set of 63 independent SNPs associated with this phenotype to test for recent positive selection, and to infer evolutionary trajectories from the ancestral versus derived status of high BMD associated alleles. These authors found statistically higher frequencies of high BMD alleles among individuals from sub-Saharan Africa, an excess of derived, low BMD alleles among Europeans and East Asians, and evidence of non-neutral evolution for BMD-associated loci by five of six tests. These findings are suggestive of selection for reduced BMD in association with human movement out of Africa, although the selective pressures remain unknown.

These studies, though limited in scope, suggest that BMD has been subject to selection in human evolution, and that tradeoffs involved in OA and OP could both be alleviated by reducing the deleterious effects of recent changes in human behavior on developing bone strength and joint flexibility. The success of such preventatives should depend on the nature of gene-by-environment interactions underlying risks of both OA and OP, and possible genetically based tradeoffs between bone strength and joint flexibility, both of which will require further study. Moreover, the degree to which novel environments actually influence the presence and strength of tradeoffs between OA and OP risk remains unclear, given the strong and diverse evidence for inverse comorbidity described earlier.

## CANCER VERSUS NEURODEGENERATIVE DISEASES

Cancer represents a large set of diseases, affecting most tissues in the body, that are unified by the presence of uncontrolled cell proliferation. Among adults, cancer risks increase sharply with age, especially after about age 50, in association with the accumulation of the multiple mutations and epimutations, within specific cell lineages, that represent its primary cause [290]. In contrast to the increased cell proliferation that typifies carcinogenesis, neurodegeneration represents increased rates of cell death within a specific, terminally differentiated cell type, neurons [291, 292]. For each neurodegenerative disease, neuronal cell death is restricted to, or concentrated in, specific regions of the brain or peripheral nervous system.

A large, robust body of epidemiological evidence demonstrates inverse associations between cancer rates and neurodegenerative disorders including Alzheimer’s (AD), Parkinson’s (PD) and Huntington’s diseases (HD) (Table 4). Cancer survivors thus exhibit substantially (∼20–50%) lower neurodegenerative disease risk, and individuals with probable AD, or diagnosed PD or HD, have comparably reduced risks of cancer, by recent meta-analyses [293–296]. Such reduced risks apply to a broad range of cancers, with one notable and well-documented exception: risk of melanoma is substantially higher among PD patients, and individuals with melanoma have a higher risk of developing PD (reviews in [339, 340]). This positive association of PD with melanoma has been explained by the fact that both diseases involve pigmented cells (melanin-producing skin cells, and neuromelanin-producing neurons in the substantia nigra), such that they share pleiotropic effects from alterations to melanin-related biochemical pathways; this hypothesis is also supported by lower PD risk among individuals with darker color of the hair or skin (see [340]). Some evidence also suggests that rates of brain cancer (which is mainly due to transformation of non-neuronal cells, or metastases) are higher among patients with PD and multiple sclerosis than in controls [294], which may be mediated by variation among individuals in brain-specific upregulation of oxidative phosphorylation pathways with increased age [341]. Additional data on brain cancers in relation to neurodegenerative diseases are required to evaluate the generality, strength and causes of these associations.

**Table 4.** Findings relevant to relationship between cancer and neurodegenerative disorders

Epidemiological data	References
Recent meta-analyses have found overall decreased risks of cancers among patients with PD (RR = 0.73, 95% CI 0.63–0.83 in [293]; ES = 0.83, 95% CI 0.76–0.91 in [294]; RR = 0.63, 95% CI 0.56–0.72 in [295]; RR = 0.55, 95% CI 0.41–0.75 in [296]), patients with AD (ES = 0.32, 95% CI 0.22–0.46 in [294]; ES = 0.42, 95% CI 0.40–0.86 in [295]) and patients with HD (ES = 0.53, 95% CI 0.42–0.67 in [294]).	[293–296]
Overall cancer risk was decreased in large cohorts of patients with PD compared with that of the general population (RR = 0.88, 05% CI 0.8–1.0 in [297]; SIR = 0.88, 95% CI 0.80–0.90 in [298]; SIR = 0.86, 95% CI 0.83–0.90 in [299]). Increased cancer risk was observed for malignant melanoma, non-melanoma skin cancer and female breast cancer; these effects may be due to risk factors shared with PD and/or to ascertainment biases (increased medical care among PD patients).	[297–300]
Overall cancer risk was non-significantly decreased in men with PD both before diagnosis (OR = 0.83, 95% CI 0.57–1.21 in [301]) and after diagnosis (RR = 0.85, 95% CI 0.59–1.22 in [302]) versus the reference population. Risk of death from cancer was also non-significantly decreased in PD patients versus the reference population even after age at onset and smoking status adjustments (HR = 0.72, 95% CI 0.43–1.23 in [303]).	[301–303]
Overall cancer risk was significantly lower in patients with PD versus those without the disease (OR = 0.72, 95% CI 0.59–0.87 in [304]; Rate ratio = 0.61, 95% CI 0.53–0.70 in [305]; HR = 0.88, 95% CI 0.78–0.99 in [306]).	[304–306]
Relative risk of cancer was higher among PD patients versus age- and sex-matched controls in a Minnesotan cohort (RR = 1.64, 95% CI 1.15–2.35), even when adjusted for smoking.	[307]
Cancer risk did not differ between a cohort of PD patients and disease-free controls (RR = 0.94, 95% CI 0.70–1.30).	[308]
Cancer risk was decreased in PD patients compared with the general population in both men (SIR = 0.79, 95% CI 0.34–1.55) and women (SIR = 0.88, 95% CI 0.35–1.81), although not significantly so. Female breast cancer risk was significantly increased in PD patients versus the general population (SIR = 5.49, 95% CI 1.10–16.03).	[309]
Relative risk of all cancers combined was reduced in patients with PD (RR = 0.92, 95% CI 0.91–0.93); cancer risk was significantly decreased for 11 cancer sites and increased for six cancer sites (including breast cancer and melanoma).	[310]
AD risk was reduced in cancer survivors versus controls without cancer in four studies (HR = 0.67, 95% CI 0.47–0.97 in [311]; RR = 0.65, 95% CI 0.56–0.76 in [312]; HR = 0.341–0.400 depending on model tested in [313]; HR = 0.57, 95% CI 0.36–0.90 in [314]). Patients with probable AD, confirmed AD or dementia (except vascular dementia) had lower subsequent cancer risks versus controls (HR = 0.39, 95% CI 0.26–0.58 in [311]; RR = 0.57, 95% CI 0.49–0.67 in [312]; HR = 0.338–0.391 depending on model tested in [313]; HR = 0.31, 95% CI 0.12–0.86 in [314]).	[311–314]
The incidence of cancer was significantly lower among patients with HD in two studies (SIR = 0.6, 95% CI 0.5–0.9 in [315]; SIR = 0.47, 95% CI 0.38–0.58 in [316]) compared with controls.	[315, 316]

Cellular, molecular and genetic data	References
*PIN1* gene, which is involved in cell proliferation and survival, is overexpressed in cancer but reduced in AD.	[317–322]
*APOE4* allele greatly increases the risk of AD but may have protective effects against cancer.	[323–325]
*TP53* gene upregulation suppresses cancer proliferation, and this gene is inactivated up to 50% of cancers. *TP53* upregulation promotes cell apoptosis, and elevated levels of p53 have been found in mice models and in the brains of patients with AD, PD and HD.	[326–330]
WNT signaling pathway is upregulated in cancer cells, and loss of WNT function is associated with AD.	[318, 331, 332]
The ubiquitin-proteosome system, mainly responsible for the degradation of intracellular proteins, shows evidence of dysfunctionality in PD, AD and HD but is upregulated in cancer.	[333–336]
Gene expression profiling of colorectal cancer, in comparison to normal colonic tissue, showed downregulation in cancer of genes associated with PD, AD, HD and oxidative phosphorylation.	[337]
Meta-analysis of transcriptomic data for AD, PD and schizophrenia, in relation to lung, prostate and colorectal cancer, showed opposite gene-expression changes between these two sets of diseases.	[338]

SIR, standard incidence rate; RR, relative risk; OR, odds ratio; HR, hazard ratio; ES, effect size; CI, confidence interval.

The striking overall magnitude of inverse epidemiological associations of most forms of cancer with PD, AD and HD, and the high prevalence of both cancer and neurodegenerative diseases, has motivated considerable research effects focused on the causes of such inverse associations, and their potential for providing insights into treatment or prevention of both sets of diseases.

### Cellular and genetic evidence on causes of cancer-neurodegeneration tradeoffs

The main causes of inverse associations between cancer and neurodegeneration trace to two characteristic features of neurons, compared with other types of cell. First, almost all neurons are long-lived and post-mitotic. Other cell types commonly enter a state of so-called cellular senescence after sufficient DNA damage or upon reaching their replicative Hayflick limit set by telomere length; such cells retain beneficial functions, but, at least in the short term, exhibit a reduced likelihood of contributing to carcinogenetic transformation [342, 343]. By contrast, neurons either repair oxidative damage more or less completely, or, if the damage is relatively severe, they undergo apoptosis, leading to cell loss. The irrevokable and deleterious nature of cell loss in otherwise long-lived neurons may thus tip cellular pathways away from apoptosis, which pleiotropically increases cancer risk in other tissues [344]. Conversely, a genetically based higher tendency toward apoptosis in neurons would reduce cancer risks but promote neurodegeneration. Such effects appear to be mediated, in part, by pleiotropic effects of variation in thresholds for apoptosis or cellular senescence, relative to retention of physiologically active cells [345].

Second, neurons exhibit high energy requirements compared with other cells, and rely primarily on oxidative phosphorylation, rather than glycolysis, to meet their elevated energetic demands. This energy-related specialization can exacerbate the accumulation of oxidative damage with age, which leads to impaired mitochondrial energy production, upregulation of oxidative phosphorylation (since glycolysis is weak), further DNA damage, and accelerating rates of neuronal apoptosis [344]. This process has been recognized as an inverse of the ‘Warburg effect’ that characterizes most cancers, which rely for energy predominantly on glycolysis [346–348]. Genetically based tradeoffs between energetic reliance on glycolysis compared with oxidative phosphorylation, and variation in thresholds for their use in the energy metabolism of cells, may therefore mediate inverse associations between cancer and neurodegeneration. This energy-based hypothesis is further supported by observations that the two populations of neurons especially highly affected by neurodegeneration, hippocampal and default-mode network neurons in AD, and neurons within the substantia nigra in PD, demonstrate relatively high energy requirements compared with other neurons [349, 350].

Several specific genes and molecular pathways have been identified as exhibiting diametric associations between cancer and neurodegenerative diseases, some of which link directly with the considerations described earlier. The gene *PIN1*, for example, codes for a protein that mediates cell proliferation and cell survival through effects on protein folding. A functional promoter polymorphism in this gene shows an inverse genotypic association with risk of AD compared with cancer [320, 321], with increased gene expression in cancer and decreases in AD. Similarly, the *APOE4* allele, which represents a strong risk factor for AD, shows evidence of negative association with risk of cancer [324]. The tumor-suppressor gene *TP53*, which directly controls tradeoffs between apoptosis, cellular senescence and cancer [329, 351], is deleted or downregulated in most cancers, but demonstrates increased expression in brains of subjects with AD [328, 352]. Two signaling pathways, the WNT pathway and the ubiquitin-proteasome pathway, also show opposite alterations to activation in cancer compared with neurodegenerative disease [331, 353, 354]. Finally, a meta-analysis of transcriptomic data for AD, PD and schizophrenia, in relation to lung, prostate and colorectal cancer, showed clear evidence of opposite gene-expression changes between these two sets of diseases [338]. These opposite alterations provide evidence that molecular tradeoffs mediate the inverse epidemiological links between cancer and neurodegeneration, in the context of the unique cellular phenotypes of neurons as well as pleiotropic effects from genes and signaling pathways that are fundamentally important in all types of cell.

Despite the clear tradeoffs underlying diametric risks of cancer and neurodegeneration, both sets of diseases are strongly age-related and driven, ultimately, by accumulation of damage to DNA and other cellular components [347, 355]. Most importantly for disease etiologies, what differs between neurons and cancer cells is the opposite nature of responses to such damage: in neural regions subject to neurodegenerative diseases, the response is apoptosis; by contrast, among incipient cancer cells, it is genetically based abrogation of apoptotic machinery combined with uncontrolled replication [344]. Such divergent effects are seen from some large-effect germline mutations in genes regulating cell proliferation: for example, losses of function in the gene *PARK2* lead to dysregulated cell cycle entry that in neurons (which are post-mitotic and incapable of replication) causes cell death but in non-neuronal cells promotes uncontrolled cell replication and carcinogenesis [356].

The common causes of aging, neurodegeneration and cancer are also seen clearly in syndromes of premature aging, which are caused by losses of DNA-repair function [357], and in the reduced cancer incidence and severity found among healthy centenarians, who presumably exhibit reductions in accumulation of damage to DNA [358]. DNA repair deficiencies in neurons have indeed been linked with a broad suite of neurodegenerative diseases [359, 360], and represent an additional causal factor mediating risks of cancer and neurodegeneration. Such positive influences on risks for both sets of diseases, from variation in overall levels of DNA damage, must however be considerably less important than diametric molecular and physiological effects, to account for the strongly inverse epidemiological patterns between cancer and neurodegeneration found across many studies.

### Evolutionary considerations and clinical implications

Alzheimer’s and Parkinson’s appear to both represent human-specific diseases [350, 361], such that evolutionary changes specific to the human lineage have potentiated their risks. The anatomical coincidence of primary Alzheimer’s-related neurological effects with the human default mode brain network [362, 363], which subserves stimulus-independent thought [89], suggests that a human-specific high metabolic rate within this region may thus underlie liability to AD [364]. Similarly, human brain evolution has been characterized by expansion in size and importance of the dopaminergic system [365], which is expected to have generated risk for neurodegeneration of dopaminergic neurons, such as those in the substantia nigra, that are especially highly active and therefore more prone to damage from oxidative stress [350]. These hypotheses provide potential explanations for why humans exhibit neurodegeneration in these two principal manifestations, and they suggest that genes and pathways modulating risks for AD and PD have been subject to positive selection in human evolutionary history [366].

The primary clinical and research-related implications of inverse associations between neurodegeneration and cancer derive from application of research findings across these two domains of disease, such that causes of one set of diseases can be considered as possible protective or therapeutic agents for the other [311, 367, 368]. Of particular importance would be situations where therapies for one disorder may be expected to increase risk for the other; for example, cancer chemotherapy has been demonstrated to increase risk of AD and other forms of neurocognitive impairment, by increasing rates of neuronal loss through apoptosis or other mechanisms [359, 369, 370]. Similarly, treatments or preventatives for neurodegenerative disorders might be expected to increase risks of cancer, unless they focused on reducing rates of the DNA damage that mediates risks of both sets of diseases. Dovetailing of results from GWAS analyses of neurogenerative diseases with those from relatively common cancers would be an especially effective means to identify shared primary causes, and diametric genetic mechanisms and pathways, that underlie risk.

## INFECTIOUS DISEASE VERSUS AUTOIMMUNITY

Autoimmunity represents the mounting of an immune reaction against one’s own cells and tissues. Such reactions are mediated by several causes, including dysregulated development of self-tolerance and self-foreign antigen recognition during early development, and excessive and overly prolonged inflammatory responses [371]. The precise mechanisms instigating autoimmunity are largely unknown, although most autoimmune disorders show high to moderate heritability and many risk alleles have been well validated [372]. With regard to elevated inflammation in autoimmunity, it is important to recognize that inflammatory responses to pathogens normally result in ‘collateral damage’ to one’s own tissues, as part of the immune response [373]. As such, levels of inflammation, and self-foreign recognition thresholds, should be subject to tradeoffs between (i) efficacy in recognition and clearance of pathogens and (ii) degrees of damage to one’s own tissues [374, 375]. Indeed, some level of autoimmune (autoantibody) response appears to provide protection against malaria [376, 377].

Infectious disease represents one of the most powerful and pervasive selective pressures impacting the human genome [378, 379]. Haldane [380] first suggested that such strong selection from disease could maintain resistance alleles even if such alleles exert deleterious effects in other contexts, such as sickle cell anemia in the classic case. By this general reasoning, strong selection from infectious disease risks may exert deleterious secondary effects largely through increasing risks of autoimmune diseases, if the effectiveness of pathogen defense trades off with risk of immune reactions against one’s own cells.

Evidence for risk of autoimmune disorders trading off with risk from infectious disease comes from three interconnected lines of research. First, a notable number of immune-system locus alleles are known that increase risks of one or more autoimmune diseases as well as protecting against one or more infectious diseases; inversely, some alleles that are protective against autoimmunity mediate increased risk of infectious disease (Table 5). In some cases, such tradeoffs are known to be driven by the effects of the alleles on expression levels of proinflammatory and anti-inflammatory cytokines [388]. Second, many risk alleles for autoimmune diseases have been demonstrated, with molecular-genetic data, to be subject to strong positive selection in humans [375, 379, 384, 395–397]. In such cases, the ‘autoimmunity risk’ alleles have apparently been favored over evolutionary time scales because they confer protection from infectious disease. The human leukocyte antigen (HLA) region 8.1 haplotype, a 4.7 Mb region found in ∼15% of Caucasians, represents an important example of such pleiotropy involving protection from infectious diseases and increased risk of a suite of autoimmune disorders, for a locus that appears to have been subject to strong positive selection [390–393]. Third, several studies have shown geographic associations of autoimmune risk alleles with higher pathogen diversity [382, 395, 398], or higher frequencies of proinflammatory (and other defense-related immune-system) alleles among individuals of tropical, compared with temperate, ancestry [399, 400].

**Table 5.** Evidence regarding diametric genotypes and phenotypes between infectious disease risk and autoimmunity

Locus or phenotype	Patterns in infectious disease and autoimmunity	References
Tumor necrosis factor gene 308A/G and 238A/G polymorphisms (proinflammatory cytokine gene)	308A allele increases risk of rheumatoid arthritis, systemic lupus erythematosis, Sjogen’s syndrome (autoimmune disorders); G allele associated with increased risk of tuberculosis; 238A allele protects from autoimmune disorders but increases risk of tuberculosis	[381]
*HLA B27* alleles (HLA-B locus family of alleles)	These alleles are associated with increased risk of ankylosing spondylitis and Reiter’s syndrome (autoimmune disorders), and with increased resistance to hepatitis C virus, and to HIV virus progression; malaria inversely associated, geographically, with prevalence of B27 and prevalence of some autoimmune disorders	[382, 383]
*SH2B3* gene (codes for adaptor protein that regulates cytokine signaling)	Alleles associated with increased risk of celiac disease also appear to confer resistance to bacterial infections	[384, 385]
*CTLA-4* gene (codes for receptor expressed by T cells) 49A/G polymorphism	A allele associated with higher risk of viral and parasitic diseases, also increases resistance to autoimmune disorders	[386]
*FUT2* gene polymorphism (null allele)	AA genotype associated with higher resistance to Norovirus, and slower HIV progression; this genotype also associated with higher risk of Crohn’s disease and Type 1 diabetes	[387]
*FOXO3A* SNP rs12212067 that modulates inflammation	G allele associated with milder Crohn’s disease and rheumatoid arthritis but with increased risk of severe malaria	[388]
*FCGR2B* gene null allele	A null allele strongly associated with higher risk of systemic lupus erythematosis also decreases risk of cerebral malaria; allele protective against malaria is more common in areas with high rates of malaria	[389]
8.1 ancestral haplotype, in HLA region	8.1 haplotype strongly associated with higher rates of wide range of autoimmune disorders; this haplotype also shown to protect against sepsis and other bacterial infections	[390–393]
*HLA-C* expression levels	Higher expression protects from HIV infection but also increases risk of Crohn’s disease	[394]
Antiautoantibody production during malaria infection	Antiautoantibody production during malarial infection appears to protect against this disease	[377]

Considered together, these three lines of evidence convergently support a hypothesis of strong, genetically based tradeoffs between protection from infectious disease and risks for autoimmune disorders, leading to diametric risks. An important caveat with regard to such tradeoffs, however, is that risk for expression of autoimmune diseases also depends notably upon whether an individual develops in the ecological environment to which their ancestors were adapted, or, by contrast, in an evolutionarily novel, more hygienic ecology [401, 402]. Thus, by the well-supported ‘hygeine hypothesis’, immune system development is more-or-less adaptively modulated and regulated by patterns of early-life exposure to parasites, commensals and mutualists; development in evolutionarily novel environments that lack such exposures may result in an ‘overactive’ immune system with the infectious-autoimmune tradeoff skewed toward autoimmunity [375, 402]. For example, risks of the autoimmune disorder systemic lupus erythematosis are on the order of 6–8 times greater for women of African descent living in the developed world than for women in Africa [403], and risks for multiple sclerosis are likewise substantially increased among individuals of African or Asian descent who were born in UK [404]. These findings indicate that infectious-autoimmune tradeoffs are exacerbated by genetically based immune-system mismatches to novel, more-hygienic environments (notably, for individuals from regions whose microbial ecologies are most similar to those of human ancestral environments), such that gene by environment interactions play important roles in tradeoff expression and effects. A primary implication of such findings is a strong case for preventatives and treatments for autoimmune diseases, and research designs to study autoimmunity, that take careful account of gene by environment interactions, environmental novelty and genetic ancestry.

A final dimension of the interface between autoimmunity and infection risk is sex differences: almost all autoimmune diseases show strong female biases in prevalence [405], which may be due, at least in part, to more robust and reactive immune systems among females than males [406]. By contrast, infectious diseases tend to be male-biased in incidence and severity [407, 408]. Determining the degree to which such sex differences are indeed due to by immune-system tradeoffs should also provide new insights into the prevention and treatment of autoimmune and infectious diseases.

## DISCUSSION

**Figure 3.**
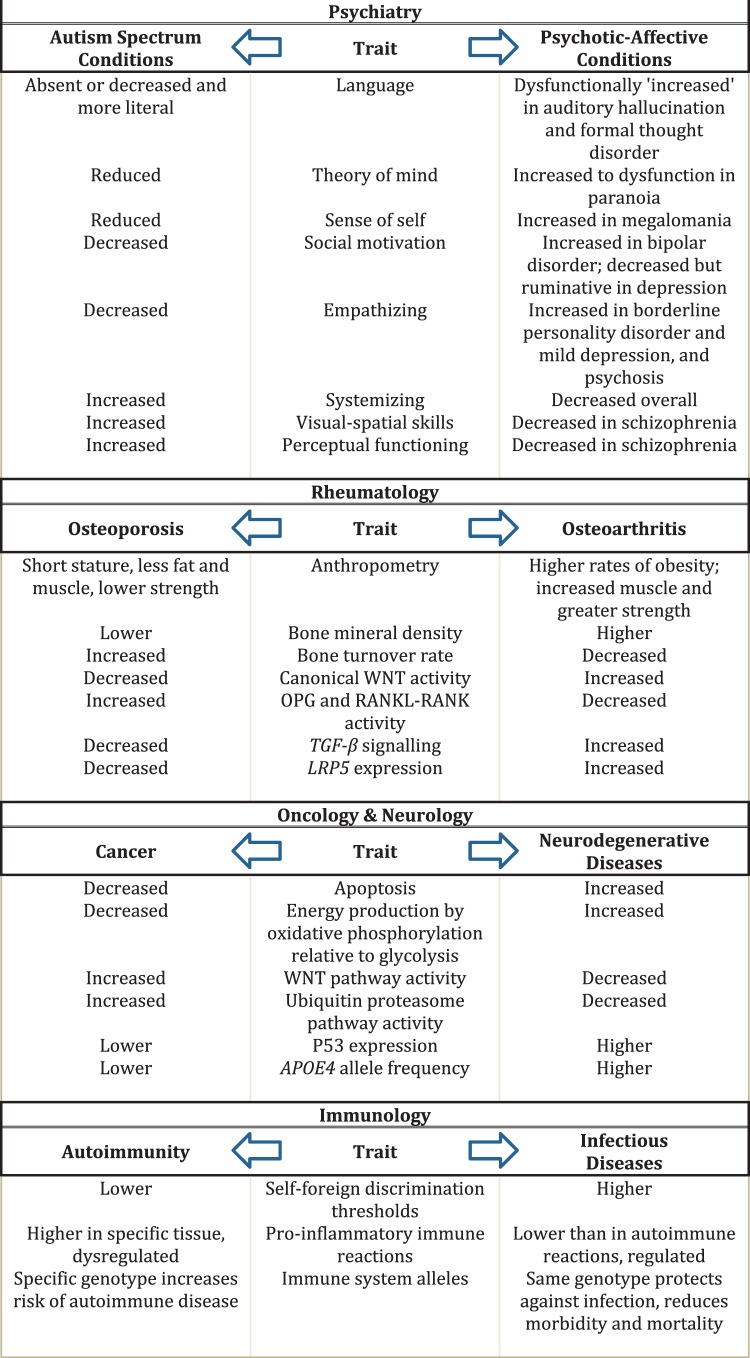
Overview of major diametric phenotypes, across the four domains of disease analysed here. See text and other tables for details. For psychiatry, this table focuses mainly on psychological phenotypes

We have described epidemiological, genetic and molecular evidence, from four disparate medical domains as summarized in Fig. 3, that suites of major human diseases exhibit diametric causes. As such, these diseases can usefully be considered in two contexts: as manifestations of relative extremes as well as failures of adaptations and as reflecting, in part, maladaptive consequences of tradeoffs between opposing optimal functions and selective pressures. The primary importance of these results is that they demonstrate substantial roles for tradeoffs and diametric effects in human polygenic disease risks, and they may direct research and clinical efforts along novel, productive paths that would not otherwise be recognized as such.

Each of the four areas of inverse disease associations described here has been postulated or discussed previously, but the conceptual commonalities between them, and their broadly applicable nature, are described here for the first time. Diametrical disorders have at their core the intrinsically bidirectional nature of biological processes, whereby expression or activation can be increased or decreased from some contextually dependent optimal value. Such processes include variation in gene expression modulated by alternative alleles at a locus, variation up or down in overall pathway activation due to single-locus, epistatic or pleiotropic effects, variation in developmentally regulated sizes of tissues or organs that develop from more or less shared precursors, and variation in development or function of major body systems, such as the immune system, hematopoetic system or brain, along major axes of discrete, microscopic or macroscopic function.

In this general context, the presence, nature and strength of tradeoffs may often depend upon both individual condition, and the degree to which increases in one phenotype necessitate decreases in another, due to pleiotropy, functional dependencies, other constraints on global optimization or degrees of homeostatic control. For example, high risks of OP or OA may only obtain at the low or high extremes of WNT signaling in bone tissue, due to some combination of genetic variation, gene by environment interactions and reduced homeostatic control. By contrast, risks for neurodegeneration compared with cancer may depend on factors that influence overall DNA damage (individual ‘condition’) as well as on how sharply an individual’s energy production and cellular-apoptotic pathways are tilted toward one set of diseases versus the other. Similarly, tradeoffs between verbal and visual-spatial skills only appear after controlling for overall IQ [21]. Tradeoffs in disease risks may thus resemble tradeoffs in evolutionary ecology, in that their expression and strength depend at least in part on overall condition and available resources [409], which are determined by both genetic and environmental contingencies. On one hand, some tradeoffs could thus be entirely precluded by ideal rearing conditions; on the other, some genetically or hormonally mediated tradeoffs may be inescapable, such that truly optimal health can never be achieved.

Recent cultural change represents an important environmental condition influencing risks of human disease, given that humans are more or less adapted to ancestral conditions [410]. Mismatches between ancestral and current conditions thus appear to be directly involved in the recent increased expression in human populations of autoimmune disorders, mediated by increases in hygiene [402], and OP, mediated by more-sedentary lifestyles [286]; longer lifespans may also increase risks of both cancer and neurodegenerative diseases, intensifying the strength of selection from both, and sharpening tradeoffs between them. Deleterious gene by environment interactions due to evolutionary mismatches may thus increase the strength of disease-related tradeoffs, to the extent that they result in diametric diseases being expressed to an increasing, and more balanced, degree, and to the extent that the diseases impact upon fitness despite their usual ages of onsets relatively late in the lifespan.

What are the expected roles of natural selection in generating and maintaining tradeoffs in disease risks? The tradeoff between infectious disease risk and risk of autoimmune diseases is clearly driven by strong positive selection for resistance to pathogens and parasites [374, 375, 384, 395–397,411], which increases the frequency of alleles with net benefits under joint selection from infections and autoimmune diseases. The importance of selection for the other three sets of diametric diseases remains more conjectural, but in each case, human-evolutionary trajectories, or ancestral selective pressures, can be plausibly inferred and empirically evaluated. Thus, human social-cognitive and affective adaptations have evolved to be much more complex than in great ape ancestors, which has apparently generated risk of psychotic-affective disorders such as schizophrenia [207] that, under the diametric model, manifest in extremes of elaborated social-brain phenotypes such as paranoia, auditory hallucinations and megalomania. Selection in this case has presumably favored ‘schizophrenia risk’ alleles, many of which also represent alleles ‘for’ complex and imaginative social cognition in neurotypical populations (B. Crespi *et al.,* submitted for publication) [412]. Risks for neurodegenerative diseases may likewise have been driven by selection for increased energetically demanding neuronal activity in particular regions of the evolving, expanding human brain, especially the default mode network and dopaminergic regions, as described earlier. Finally, the presence of healed and unhealed bone fractures in hominin fossil remains (e.g. [413–415] suggests that fractures are likely to have represented non-trivial causes of morbidity and mortality across human evolutionary history; by this reasoning, ongoing selection for strong bones would generate and increase risk of OA. Osteoarthritic damage has been commonly described from the hominin fossil record [416–419], although its quantitative incidence remains unclear. More broadly, Jurmain [283] reported significantly lower incidence of OA and osteophytosis (bone spur formation, which is associated with OA) in chimpanzees, gorillas and bonobos compared with humans, which he attributed, in part, to the obligate bipedalism of humans.

These hypotheses regarding selection pressures and evolutionary polarities, although largely speculative, can be subject to robust tests through genomic analyses that combine GWA tests for disease risk alleles with analyses of positive and balancing selection. Indeed, to the extent that one disease becomes less common over time due to selection and genetic response, its opposite would be expected to increase in frequency, a process that may tend to maintain genetic variation for disease risks in natural populations. Such studies may also be useful in evaluating the degree to which disease risks other than infectious disease, and effects of antagonistic pleiotropy, represent causes of selection in recent human evolution.

The relative degree to which major sets of human diseases exhibit inverse associations, compared with patterns of positive association in comorbidity, remains unknown, but comorbidities have been much more widely studied and appear to be more common [420]. Why should this be so? First, most diseases involve losses of function in adaptive systems, which are expected to be more likely responses to genetic or environmental perturbations than gains of function; effects of increased activation or expression may also often be prevented or ameliorated by negative feedbacks. Such losses may often affect overlapping disease-related tissues and organs. Comorbidities may also be generated when reduced function of one system increases probabilities for dysfunctions or failures of others. Second, diagnostic uncertainties, overlaps and falsely categorical diagnoses can generate comorbidities that are more apparent than real; for example, the psychotic-affective condition borderline personality disorder exhibits high comorbidity with depression, bipolar disorder, post-traumatic stress disorder and substance abuse [421], mainly because its diverse diagnostic phenotypes, causes and symptoms overlap broadly with those of many other psychiatric disorders. For such conditions, dimensional classifications along quantitative symptom axes may provide more biologically justifiable and useful frameworks for diagnostics and treatment than ones that are categorical [9].

Additional analyses of the conditions under which diseases exhibit diametric, compared with positively associated, patterns and causes, should guide further study of the roles of tradeoffs and pleiotropy in human disease. A primary benefit of focusing on the diametric nature of disease risks is that it jointly illuminates the causes of two sets of diseases as well as providing direct insights into factors that protect from risk and may serve as novel agents of therapy. For example, single loci that exhibit diametric genetic effects on risk, such as *LRP5* in OP and OA, and *PIN1* in cancer and AD, demonstrate dosage-sensitive effects on disease-relevant pathways, which typically represent good indicators of enzyme or ligand-receptor systems that are especially amenable to pharmacological intervention.

## FUNDING

The authors thank the Natural Sciences and Engineering Research Council of Canada (Grant Number 611201) for financial support.
